# TRPV2 calcium channel promotes breast cancer progression potential by activating autophagy

**DOI:** 10.1186/s12935-024-03506-y

**Published:** 2024-09-27

**Authors:** Qing Li, Huixian Li, Ruiwen Zhu, William Chi Shing Cho, Xiaoqiang Yao, Fung Ping Leung, Gary Tse, Lai Kwok Leung, Wing Tak Wong

**Affiliations:** 1 School of Life Sciences, Faculty of Science, The Chinese University of Hong Kong, Hong Kong, 999077 China; 2 State Key Laboratory of Agrobiotechnology, The Chinese University of Hong Kong, Hong Kong, 999077 China; 3 Department of Clinical Oncology, Queen Elizabeth Hospital, Kowloon, Hong Kong SAR 999077 China; 4 School of Biomedical Sciences, The Chinese University of Hong Kong, Hong Kong, 999077 China; 5 School of Nursing and Health Studies, Hong Kong Metropolitan University, Hong Kong, 999077 China; 6 Tianjin Key Laboratory of Ionic-Molecular Function of Cardiovascular Disease, Department of Cardiology, Tianjin Institute of Cardiology, Second Hospital of Tianjin Medical University, Tianjin, 300211 China

**Keywords:** Breast cancer, TRPV2, Calcium channel, Autophagy, CaMKKβ, AMPK, ULK1

## Abstract

Breast cancer, the most prevalent and aggressive tumor affecting women, requires identification of disease determinants to facilitate the development of effective therapeutic strategies. Transient receptor potential vanilloid 2 (TRPV2), an ion channel highly permeable for calcium (Ca^2+^), is implicated in physiological and pathological processes. Nevertheless, the role of TRPV2 in breast cancer remains poorly elucidated. In this study, we found high levels of TRPV2 expression associated with advanced malignancy, thereby suggesting its potential as a biomarker for breast cancer staging. We demonstrated that TRPV2 activation promotes breast cancer cell proliferation, migration, and invasion, while silencing of TRPV2 suppresses breast cancer progression, highlighting the oncogenic role of TRPV2. Moreover, we reveal that TRPV2 facilitates cancer progression by modulating the CaMKKβ/AMPK/ULK1-autophagic axis through mediating calcium influx, providing new insights into TRPV2 as a novel therapeutic target for breast cancer treatment.

**Supplementary Information:**

The online version contains supplementary material available at 10.1186/s12935-024-03506-y.

## Introduction

Breast cancer represents a commonly diagnosed malignancy and stands as the foremost cause of cancer-related mortality in women globally [1, 2]. In the past decade, the integration of genomic and transcriptomic studies has provided valuable insights into the underlying molecular drivers, clonal evolutionary trajectories, and prognostic signatures associated with breast cancer [3]. Breast cancer is classified into four subtypes, namely Luminal A, Luminal B, Her2-enriched, and triple-negative, which are determined by the expression levels of estrogen receptor (ER), progesterone receptor (PR), and ERBB2 receptor (HER2) [4, 5]. Treatment options for breast cancer, such as breast-conserving surgery or mastectomy, chemotherapy, radiotherapy, and hormone therapy, are predicated upon the evaluation of individual clinico-pathological features [6, 7]. Despite recent advancements in early diagnosis and effective treatment, the recurrence or metastasis of breast cancer continues to pose a formidable challenge to the overall survival of affected patients [8–10]. Therefore, to improve treatment outcome, it is imperative to unravel the molecular mechanisms driving breast cancer progression and identify determinants contributing to the aggressiveness of the disease.

Autophagy, as a ubiquitous and conserved process, serves as an energy recycling system that transports misfolded proteins, damaged organelles, and intracellular constituents to lysosomes for degradation [8, 11]. Dysregulation of this process can lead to various cellular pathological alterations, thereby promoting disease progression, including cancer [12–18]. In advanced cancers, the upregulation of autophagy plays a vital role in recycling cytoplasmic components and providing amino acids and ATP to support essential metabolic pathways and protein synthesis [19, 20]. Consequently, autophagy facilitates tumor growth by aiding the adaptation to environmental and metabolic stressors, such as hypoxia and starvation [21, 22]. Autophagy has emerged as a significant factor contributing to the progression of breast cancer. For instance, the activation of P2 × 4 signaling promotes autophagy and epithelial-to-mesenchymal transition, thereby driving breast cancer progression and aggressiveness [23]. Additionally, RSK2 plays a protective role in human breast cancer cells by mitigating endoplasmic reticulum stress through the activation of AMPKα2-mediated autophagy [24]. Conversely, the suppression of autophagy using small molecule inhibitors that target Vps34 augments the susceptibility of breast cancer cells to Sunitinib [25]. Consequently, understanding the underlying mechanisms that regulate autophagy in breast cancer holds great potential for developing therapeutic strategies based on autophagy modulators.

Accumulating evidence highlights the significance of intracellular Ca^2+^ in the regulation of basal autophagy and its response to stress conditions [26, 27]. Moreover, multiple studies have established a potential association between the disruption of Ca^2+^ homeostasis in the mammary gland and the onset of breast cancer, thereby positioning Calcium Transient Receptor Potential (TRP) channels as promising targets for therapeutic interventions in breast cancer [28, 29]. TRPC1 has been implicated in breast cancer cell proliferation [30], whereas TRPC6, TRPM7, and TRPV6 have demonstrated their involvement in the regulation of breast cancer cell proliferation and migration [31–35]. Moreover, previous investigations have indicated the potential utility of TRPM8 as a differentiating marker in breast cancer [29, 36].

TRPV2, a member of the TRPV family, exhibits high selectivity for Ca^2+^. Upon activation, a significant portion of TRPV2 translocates from the endoplasmic reticulum (ER) membrane to the plasma membrane. During this translocation, TRPV2 within the ER can undergo sorting into small vesicles by budding from the ER membrane. Subsequently, these vesicles are transported to the plasma membrane, where they fuse with it through exocytosis, enabling TRPV2 to function as a cation channel on the cell surface [37]. Previous studies have implicated TRPV2 in cardiac regulation and contractility involving calcium handling [38]. More recently, attention has shifted towards understanding the role of TRPV2 in tumorigenesis across various tissues. Notably, studies have linked TRPV2 expression in liver tissues with hepatocarcinogenesis [39]. In gastric cancer, TRPV2 has been shown to promote cell migration and invasion through the Transforming Growth Factor-β signaling pathway [40]. Moreover, TRPV2 has been implicated in mediating adhesion, migration, and invasion in prostate and urothelial cancer cells under the stimulation of Adrenomedullin [41]. In human melanoma, the mechanosensitive TRPV2 calcium channel has been found to enhance invasiveness and metastatic potential [42]. Activation of TRPV2 has also been associated with cancerous behaviors in esophageal squamous cell carcinoma cells [43]. While these studies suggest the potential of targeting TRPV2 as a therapeutic approach in cancer treatment, its role in breast cancer progression remains largely unexplored.

Our study provides evidence showcasing the high expression of TRPV2 in advanced stages of breast cancer, underscoring its potential as a biomarker for advanced disease. Moreover, our findings shed light on the potential therapeutic implications of TRPV2 in promoting the progressive potential of breast cancer through the modulation of autophagy via calcium signaling.

## Materials and methods

### Cell culture

We employed three breast cancer cell lines, namely MCF-7 (ER^+^, PR^+/−^, HER2^−^), SK-BR-3 (ER^−^, PR^−^, HER2^+^), and MDA-MB-231 (ER^−^, PR^−^, HER2^−^), along with a normal breast cell line, MCF-10 A human breast epithelial cells for in vitro study. MCF-7 and SK-BR-3 cells were cultured under standard conditions in Dulbecco’s modified Eagle’s medium (DMEM, Thermo Fisher, Cat# 10965092) supplemented with 10% fetal bovine serum (FBS, Thermo Fisher, Cat# A4766801) and 1% penicillin-streptomycin (PS, Thermo Fisher, Cat# 15140122) at 37 °C in a humidified atmosphere containing 5% CO_2_. MDA-MB-231 cells were cultured in Leibovitz’s L-15 medium (Thermo Fisher, Cat# 11415064) supplemented with 10% FBS and 1% PS at 37 °C, in a humidified atmosphere without 5% CO_2_. MCF-10 A cells were maintained in DMEM/F12 medium (Thermo Fisher, Cat# 121331020) supplemented with 20 ng/mL epidermal growth factor (Peprotech, Cat# AF100122), 0.5 mg/mL hydrocortisone (Sigma-Aldrich, Cat# 50237), 100 ng/mL cholera toxin (Sigma-Aldrich, Cat# 9012639), 100 mg/mL insulin (Sigma-Aldrich, Cat# 11070738), 1% PS, and 10% horse serum (Invitrogen, Cat# 16050122) at 37 °C, in a humidified atmosphere with 5% CO_2_.

### Cell proliferation assay

The MTT assay was performed to determine the proliferation rate of breast cancer cell line [44]. A total of 2 × 10^3^ cells were seeded in each well of a 96-well plate. The cells were incubated for 24, 48, and 72 h, respectively. After the respective incubation periods, 20 µL of a 5 mg/mL MTT solution (Merck, Cat# M2003) was added to each well and incubated at 37 °C for 4 h. Subsequently, the supernatant was aspirated, and 150 µL of DMSO was added to each well to dissolve the purple formazan crystals. The absorbance of the 96-well plate was then measured at 490 nm using a microplate reader.

### Colony formation assay

1 × 10^2^ MCF-7, SK-BR-3, and MDA-MB-231 cells were seed at 6-well plates, which were then incubated for a duration of 2–3 weeks. Following incubation, the complete medium was aspirated, and the cells were washed with PBS. To fix the cells, 4% paraformaldehyde (Cat. No. 158127, Sigma-Aldrich, St. Louis, MO, USA) was applied for 30 min, after which it was removed, and the cells were further rinsed with PBS. Subsequently, 1 mL of Giesma (Cat. No. 48900, Sigma-Aldrich, St. Louis, MO, USA) stain was added to each well, allowing the colonies to be stained for 30 min. Excess Giesma stain was washed away using water, and the plates were left to air dry at room temperature. The colonies were then counted under a stereomicroscope.

### Cell cycle assay

FxCycle™ PI/RNase Staining Solution (Thermo Fisher, Cat# F10797) was applied to assess the cell cycle of breast cancer cells. The cells were harvested and suspended at a concentration of 4 × 10^5^ in 5 mL of cold PBS. The cell samples were then centrifuged at 1,000 g for 5 minutes at 4 °C, and the PBS was carefully removed. Subsequently, the cells were fixed with 75% ethanol for 24 h. After fixation, the cells were centrifuged again at 1,000 g for 5 minutes at 4 °C, and the ethanol was aspirated. The cells were then washed twice with cold PBS, ensuring that all fixative solutions were thoroughly removed. Next, the cells were re-suspended in 200 µL of cold PBS and 200 µL of FxCycle™ PI/RNase Staining Solution, followed by incubation for 20 min at room temperature while protected from light. The fluorescence of the cells was then measured and analyzed at 585/540 nm using a flow cytometer.

### Wound healing assay

A total of 2 × 10^6^ MCF-7, SK-BR-3, and MDA-MB-231 cells were seeded into each well of a six-well plate and allowed to incubate for 24 h. Using 10 µL pipette tips, a 1 mm wound field was created in the center of the wells. Dead cells were subsequently washed away by replacing the medium with FBS-free complete medium. The cells were observed under a light microscope at 24-hour intervals. The wound areas at 0 h and 72 h were quantified using Image J software. The healing area was calculated by subtracting the wound area at 0 h from the wound area at 72 h. Subsequently, the recovery percentage was determined by comparing the healing area with the initial wound area at 0 h.

### Cell invasion assay

The lower compartment of the transwell was filled with medium containing 0.5% FBS and 40 µg/mL collagen. The transwell insert was carefully immersed into the lower compartment, ensuring the bottom of the insert was submerged in the medium. The upper compartment of the transwell was filled with complete medium comprising 10% FBS and 1% PS. Subsequently, a total of 1 × 10^5^ MCF-7, SK-BR-3, and MDA-MB-231 cells were collected and seeded into the upper compartment of the transwell. The cells were then incubated for 3 h, allowing for migration towards the underside of the insert filter. Cells remained on the upper side of the filter membrane were gently removed using a cotton swab. The cells on the lower side of the insert were fixed with 5% glutaraldehyde for 10 min. Following fixation, the cells were stained with 1% crystal violet in 2% ethanol for 20 min. Excess crystal violet was swiftly removed by briefly immersing the insert in ddH2O for 3 to 4 s, and the insert membrane was left to air dry at room temperature. The total number of migrated cells was then counted under a microscope.

### Ca^2+^ imaging

Breast cancer cells were cultured in confocal imaging dishes for confocal microscopy. The cultures were treated with normal physiological saline solution (NPSS) with 5 µM Fluo-4 AM (Thermo Fisher, Cat# F1420) and 0.02% pluronic acid F-127 (Thermo Fisher, Cat# P3000MP), and Incubated in the dark at room temperature for 30 min. Subsequently, the cells were washed and immersed in either NPSS (with external Ca^2+^) or OPSS (without external Ca^2+^). OPSS was prepared using the same formula as NPSS, but with 0.2 mM EGTA instead of Ca^2+^. 488 nm excitation wavelength were applied to illuminate the cells. Real-time monitoring and recording of changes in cytosolic Ca^2+^ ([Ca^2+^]_i_) fluorescence intensity were performed using a confocal microscope. The ratio of [Ca^2+^]_i_ fluorescence normalized to background fluorescence (F1/F0) was calculated. Changes in fluorescence were measured as the maximum peak response above the baseline. The control involved the use of the solvent DMSO, and the TRPV2 activator cannabidiol was added to induce Ca^2+^ influx. Ionomycin (Cayman Chemical, Cat# CAY10004974), a calcium ionophore, was used as a positive control for inducing calcium influx.

### Lentiviral production and transduction

9 × 10^6^ 293T cells were seeded in a 10mm cell cuture dish the day before lentivirus production. Lipofectamine 3000 (Thermo Fisher, Cat# L3000008) and DNA mixes were prepared accordingly. Tube A received 1.5 mL Opti-MEM medium (Thermo Fisher, Cat# 31985070) and 41 µL Lipofectamine 3000, while tube B contained 1.5 mL Opti-MEM medium, 6 µg packaging plasmid, 2 µg envelope plasmid, and 8 µg TRPV2 plasmid (Sigma-Aldrich, Cat# SHCLND-NM_016113). Tube A and tube B solutions were mixed by gentle pipetting and incubated at room temperature for 15 min to form lipid-DNA complexes. The complexes were then carefully added to cell culture dish, avoiding cell dislodgment, and incubated at 37 °C with CO_2_. After 12 hours, the transfection medium was replaced with 10 mL of warm medium. The medium containing the virus was harvested 48 h later and filtered using a 0.22 μm Steriflip (Merck, Cat# SE1M179M6) prewetted with 500 µL PBS. Following filtration, the filtered viral supernatant was mixed with cold Lentivirus Precipitation Solution (ALSTEM, Cat# VC100) and centrifuged at 1500 g for 30 min at 4 °C, resulting in a beige pellet of lentivirus. The supernatant was carefully removed, and the lentiviral pellets were resuspended in 100 µL of serum-free medium. Subsequently, 1 × 10^6^ MCF-7, SK-BR-3, and MDA-MB-231 cells were seeded into separate six-well plates and incubated at 37 °C with CO_2_. After 24 h, the transfection medium was prepared by adding 12 µL of 10 mg/mL polybrene (Santa Cruz Biotechnology, Cat# SC-134220) to 15 mL of complete growth medium. 2 mL of the transfection medium and 20 µL of concentrated virus were added to each well of the six-well. The plates were incubated overnight at 37 °C with CO_2_, followed by a change to fresh growth medium containing 10 mg/mL polybrene. Antibiotic selection ensured cell survival, and the phenotype was evaluated using Western blot analysis over the subsequent days.

### RNA extraction and quantitative PCR (qPCR)

RNA isolation from cultured cells was performed using RNAi Plus (Takara, Cat# 9108) following the manufacturer’s instructions. The isolated RNA was then dissolved in DEPC-treated water, and its purity was assessed. PrimerScriptTM RT Master Mix (Takara, Cat# RR036A) was applied to obtained cDNA by reverse transcription [45]. Then, Real-time PCR was carried out using TB Green Premix Ex Taq II (Takara, Cat# RR820A). PCR reactions were set up according to the manufacturer’s protocol, and the transcriptional expression level was normalized to the GAPDH internal control. The data were analyzed using the delta-delta CT method.

### Western blot

Cells were washed twice with cold PBS. Subsequently, cold RIPA Lysis Buffer (Thermo Fisher, Cat# 89900) containing protease inhibitors (Thermo Fisher, Cat# 78410) was added to the cells, followed by incubation on ice for 30 min. The lysate was then collected and centrifuged at 12,000 g for 15 min at 4 °C, and the supernatant was transferred to a new tube for protein concentration analysis. The protein concentration was determined using the Thermo Scientific™ Pierce™ BCA Protein Assay Kit (Thermo Fisher, Cat# 23225). For protein separation, 10 − 12.5% SDS-PAGE was performed, and after electrophoresis, the protein samples were transferred onto 0.22 μm PVDF membranes. Then, the membrane was incubated with the primary antibodies overnight at 4 °C. Subsequently, HRP-conjugated secondary antibodies were incubated for 60 min to enable chemiluminescent protein detection. Protein intensity was quantified using ImageJ software.

### Immunofluorescence

Cover glasses were placed in 24-well plates, and a total of 1 × 10^5^ MCF-7, SK-BR-3, and MDA-MB-231 cells were seeded into each well. The plates were then incubated at 37 °C in a humidified atmosphere with 5% CO_2_ for 24 h. Subsequently, the cells were washed twice with PBS and fixed with 4% paraformaldehyde at room temperature for 15 min. To permeabilize the cells, 0.1–0.5% Triton X-100 was added to the cells for 10 min. Next, the cells were incubated with 10% normal goat serum at room temperature for 30 min. Primary antibodies were applied to the cells and incubated overnight at 4 °C. Then, Fluorochrome-conjugated secondary antibodies were added to the cells and incubated at 37 °C in the dark for 60 min. To stain the nuclei, 1 µg/mL DAPI (Thermo Fisher, Cat# 62247) was applied to the cells for 5 minutes at room temperature. Finally, a drop of mounting medium was used to mount the slides, and the fluorescence was observed using confocal microscopy.

### Immunohistochemistry (IHC)

Following dissection, tumor samples were fixed using 10% Formalin (Sigma-Aldrich, Cat# 110735). The fixed samples were embedded in paraffin and sectioned into 4 μm. To remove the wax, the slides were immersed in xylene for 10 min and then rehydrated by passing through a series of graded alcohol solutions. Endogenous peroxidase activity was then quenched using 3% hydrogen peroxide. The sections were subsequently blocked using 10% normal goat serum for 1 h. Primary antibodies were applied and incubated at 4 °C overnight, followed by incubation with secondary antibodies for 1 h. Signal detection was achieved using DAB (Dako, Cat# K4003).

### Animal model

4-week-old female Balb/c nude mice were purchased from CUHK University Laboratory Animal Services Center. MDA-MB-231 cells, which had been transfected with either lentiviral small hairpin RNAs (shRNAs) (shTRPV2-1 or shTRPV2-2) or overexpression plasmid (OE-TRPV2), were harvested and suspended in 100 µL of sterile PBS within an Eppendorf tube. This cell suspension was then mixed with 100 µL of thawed Matrigel. Subsequently, the cancer cells were carefully loaded into an insulin syringe and injected into the fourth left mammary fat pad of the nude mice. Anesthesia was administered to the mice using a combination of 80 mg/kg ketamine, 2 mg/kg acepromazine, and 5 mg/kg xylazine when the tumor volume of the control group reached 1,000 mm^3^. Following the induction of anesthesia, the mice were euthanized, and the tumors were excised for subsequent immunohistochemistry staining and immunofluorescence staining.

### Statistical analysis

Statistical analysis was performed using GraphPad Prism 9, and the data were presented as mean values with the corresponding standard error of the mean (SEM). Multiple sample comparisons were analyzed using one-way ANOVA followed by Dunnett’s Multiple Comparison Test. Two-group comparisons were conducted using paired student t-tests. The significance level for all statistical analyses was set at ∗*p* < 0.05. ∗∗*p* < 0.01, ∗∗∗*p* < 0.001 are indicated.

## Results

### TRPV2 is highly expressed in breast cancer and indicates advanced cancer stage

**Fig. 1:**
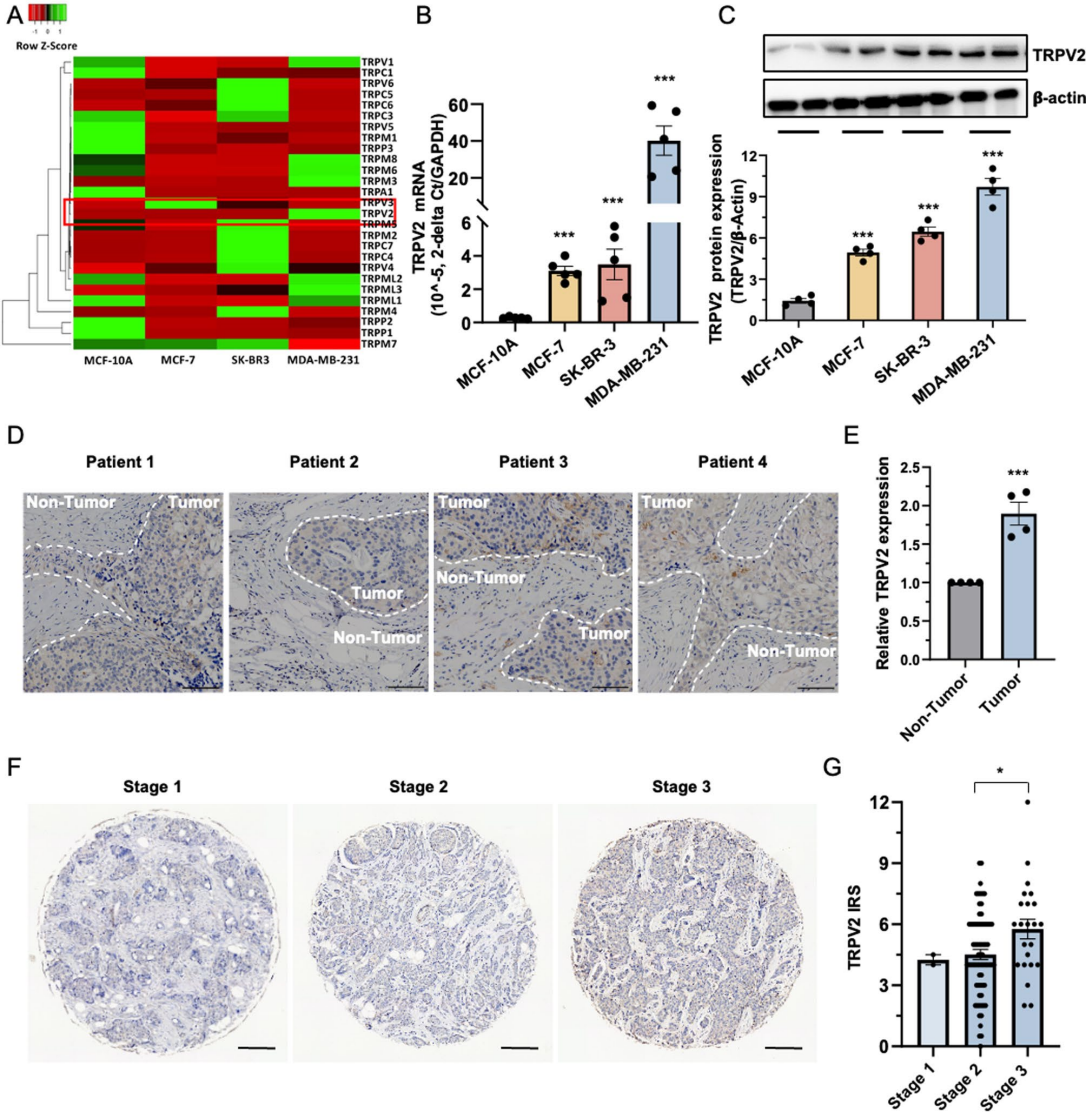
Elevated expression of TRPV2 in breast cancer and its association with advanced cancer stage. **(A)** Comparative transcriptional analysis of 27 TRP channels in normal breast epithelial cells (MCF-10 A) and three breast cancer cell lines (MCF-7, SK-BR-3, and MDA-MB-231) (*n* = 8). **(B)** Expression of TRPV2 transcript in a normal breast epithelial cell line (MCF-10 A) and three breast cancer cell lines (MCF-7, SK-BR-3, and MDA-MB-231) (*n* = 5). **(C)** Representative immunoblots (upper panel) and quantification (lower panel) of TRPV2 protein expression in a normal breast epithelial cell line (MCF-10 A) and three breast cancer cell lines (MCF-7, SK-BR-3, and MDA-MB-231) (*n* = 4). **(D** and **E)** Representative image **(D)** and quantification **(E)** of IHC staining showing TRPV2 expression in breast tumor and adjacent non-tumor of triple-negative subtype from breast cancer patient (TMA# BR1505C) (*n* = 4; scale bar, 100 μm). **(F** and **G)** Representative image **(F)** and quantification **(G)** of IHC staining showing TRPV2 expression in breast tumor samples from patients in the tissue microarray cohort (BR1902) (*n* = 95; scale bar, 50 μm). Error bar represents mean ± SEM. *p < 0.05, ***p < 0.001; Student’s t test in **(D-E)**, analysis of variance test (ANOVA) in **(B-C** and **F-G)**. TRP, transient receptor potential; IRS, immunoreactive score

To investigate the potential involvement of TRP channels in breast cancer, a comparative analysis was conducted to assess the expression of 27 TRP channels in the normal breast epithelial cell line, MCF-10 A, as well as three breast cancer cell lines (MCF-7, SK-BR-3, and MDA-MB-231). RT-PCR analysis revealed an overexpression of TRPV2 in the breast cancer cell lines compared to normal breast cells (Fig. 1A-B). We further employed Western blotting to evaluate the protein expression of TRPV2 in these four cell lines. Consistently, TRPV2 expression exhibited a significant increase in MCF-7, SK-BR-3, and MDA-MB-231 cells in comparison to MCF-10 A cells (Fig. 1C). Notably, our analysis also revealed a differential expression of TRPV2 in breast cancer cells. Specifically, we observed a decreased expression of TRPV2 in the MCF-7 cell line, characterized by low metastatic potential. Conversely, the high metastatic breast cancer cell line, MDA-MB-231, demonstrated higher expression of TRPV2 (Fig. 1C). These findings suggest a potential positive correlation between TRPV2 expression and the metastatic ability of breast cancer cells.

To further validate our findings, we conducted IHC staining to examine TRPV2 expression in breast cancer tissues obtained from patients. Interestingly, we observed higher levels of TRPV2 expression in the tumor area compared to adjacent non-tumor tissue (Fig. 1D-E). Subsequently, we conducted an analysis on a cohort of breast cancer patients using a tissue microarray (TMA#BR1902) comprising samples from various stages of the disease. The analysis was stratified based on TRPV2 expression levels in the tumor samples. When considering the whole cohort, we observed lower TRPV2 expression in stage 1 and stage 2 tumors, while higher TRPV2 expression was evident in more advanced tumor stages, specifically in stage 3 (Fig. 1F-G). Collectively, these findings imply a potential involvement of TRPV2 in the progression of breast cancer, suggesting a significant role of TRPV2 in the disease advancement.

### Silencing of TRPV2 impedes the proliferation and metastasis of breast cancer cells

To investigate the role of TRPV2 in breast cancer cells, we employed a small interfering RNA (siRNA) targeting TRPV2 (SiTRPV2) in MCF-7, SK-BR-3, and MDA-MB-231 cell lines. The efficacy of siRNA interference was determined by assessing TRPV2 expression through qPCR and WB analysis. Our findings demonstrated a significant reduction in TRPV2 mRNA levels in breast cancer cells (MCF-7, SK-BR-3, and MDA-MB-231) transfected with TRPV2 siRNA (Figure S1A). Moreover, the siTRPV2 groups exhibited decreased protein expression of TRPV2 compared to the normal control groups (Figure S1A-B). These results indicate the successful downregulation of TRPV2 using siRNA, providing a valuable model for studying the biological function of TRPV2.

**Fig. 2:**
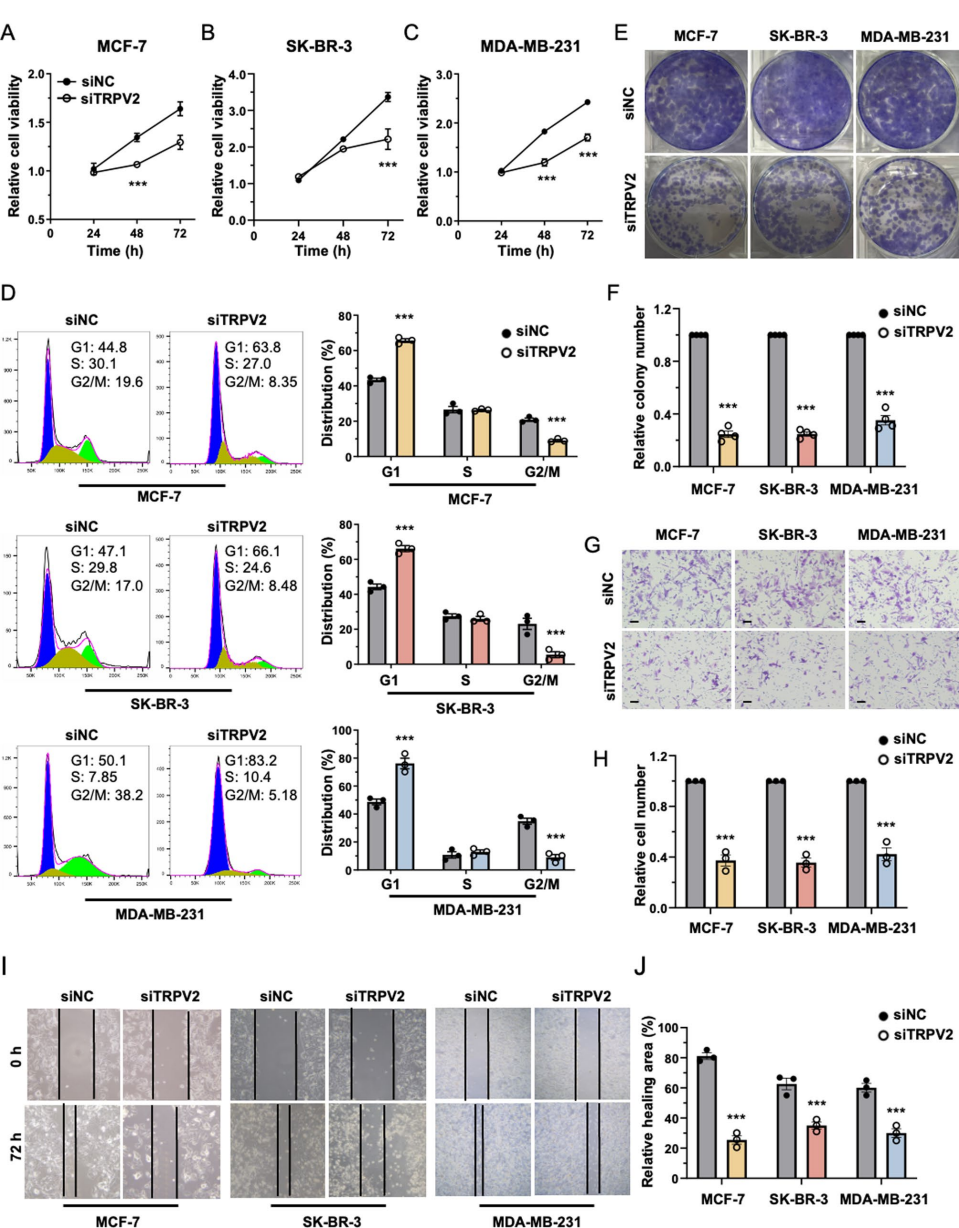
Inhibition of TRPV2 hinders the proliferation and metastasis of breast cancer. **(A-C)** Quantification of cell viability in MCF-7 **(A)**, SK-BR-3 **(B)**, and MDA-MB-231 **(C)** breast cancer cells with or without TRPV2 knockdown by MTT assay (*n* = 6). **(D)** Representative flow plot (left) and quantitative analysis (right) depicting the cell cycle distribution in MCF-7, SK-BR-3, and MDA-MB-231 cells with or without TRPV2 knockdown (*n* = 3). **(E-F)** Representative image **(E)** and quantification **(F)** of colony formation in breast cancer cells with or without TRPV2 knockdown (*n* = 4). **(G-H)** Representative image **(G)** and quantification **(H)** of invaded cells in breast cancer cells transfected with si-NC or si-TRPV2, assessed using the transwell assay (*n* = 3; scale bar, 10 μm). **(I-J)** Representative image **(I)** and quantitative analysis **(J)** of the wound healing area in breast cancer cells with or without TRPV2 knockdown (*n* = 3). Error bar represents mean ± SEM. ***p < 0.001; Student’s t test in **(E-J)**, analysis of variance test (ANOVA) in **(A-D)**. NC, negative control

We then employed the MTT assay to examine the impact of TRPV2 on breast cancer cell proliferation. Knockdown of TRPV2 in MCF-7, SK-BR-2, and MDA-MB-231 breast cancer cells resulted in diminished cell growth (Fig. 2A-C). Moreover, we performed flow cytometry analysis to evaluate the influence of TRPV2 on the cell cycle. Remarkably, TRPV2 knockdown impeded cell cycle progression, as evidenced by an increased proportion of cells in the G1 phase and a decreased proportion in the G2 or M phase (Fig. 2D). Consistent with these observations, silencing of TRPV2 suppressed colony formation in MCF-7, SK-BR-3, and MDA-MB-231 breast cancer cells (Fig. 2E-F). Subsequently, we employed transwell assay to investigate the impact of TRPV2 on the invasion of breast cancer cells in vitro. Our data revealed that TRPV2 exerts a promotional effect on breast cancer cell invasion, as evidenced by a significant reduction in the number of invaded cells upon TRPV2 silencing (Fig. 2G-H). Furthermore, we explored the effects of TRPV2 on breast cancer cell migration using a wound healing assay. Notably, at the 72-hour time point, a reduction in the relative healing area was observed in MCF-7, SK-BR-3, and MDA-MB-231 breast cancer cells transfected with TRPV2 siRNA compared to the control group (Fig. 2I-J). Collectively, these findings support the oncogenic role of TRPV2 in the growth and metastasis of breast cancer.

### Activation of TRPV2 facilities the proliferative and metastatic potential of breast cancer cells

Given the high expression of TRPV2 in cancer cells (Fig. 1C), our subsequent investigation aimed to determine whether the activation of TRPV2 contributes to cancer progression using cannabidiol, a TRPV2 activator. Our results demonstrated that cannabidiol significantly increased both the mRNA level (Figure S2A) and protein level (Figure S2B-C) of TRPV2 in MCF-7, SK-BR-3, and MDA-MB-231 breast cancer cells. Furthermore, the activation of TRPV2 by cannabidiol exhibited increased proliferation of MCF-7, SK-BR-3, and MDA-MB-231 breast cancer cells, as evidenced by our MTT assay (Figure S3A-C). Additionally, cannabidiol treatment induced a notable shift in the cell cycle, with an increased proportion of cells in the G2 phase (Figure S3D). Consistently, cannabidiol promoted colony formation in the aforementioned breast cancer cell lines (Figure S3 E-F). Moreover, cannabidiol enhanced the invasion of MCF-7, SK-BR-3, and MDA-MB-231 breast cancer cells in vitro (Figure S3G-H). Notably, cannabidiol significantly increased the relative healing area in MCF-7, SK-BR-3, and MDA-MB-231 breast cancer cells, as demonstrated by our wound healing assay (Figure S3 I-J). Collectively, these findings suggest that the activation of TRPV2 promotes breast cancer progression.

### TRPV2 mediates calcium influx in breast cancer cells

**Fig. 3:**
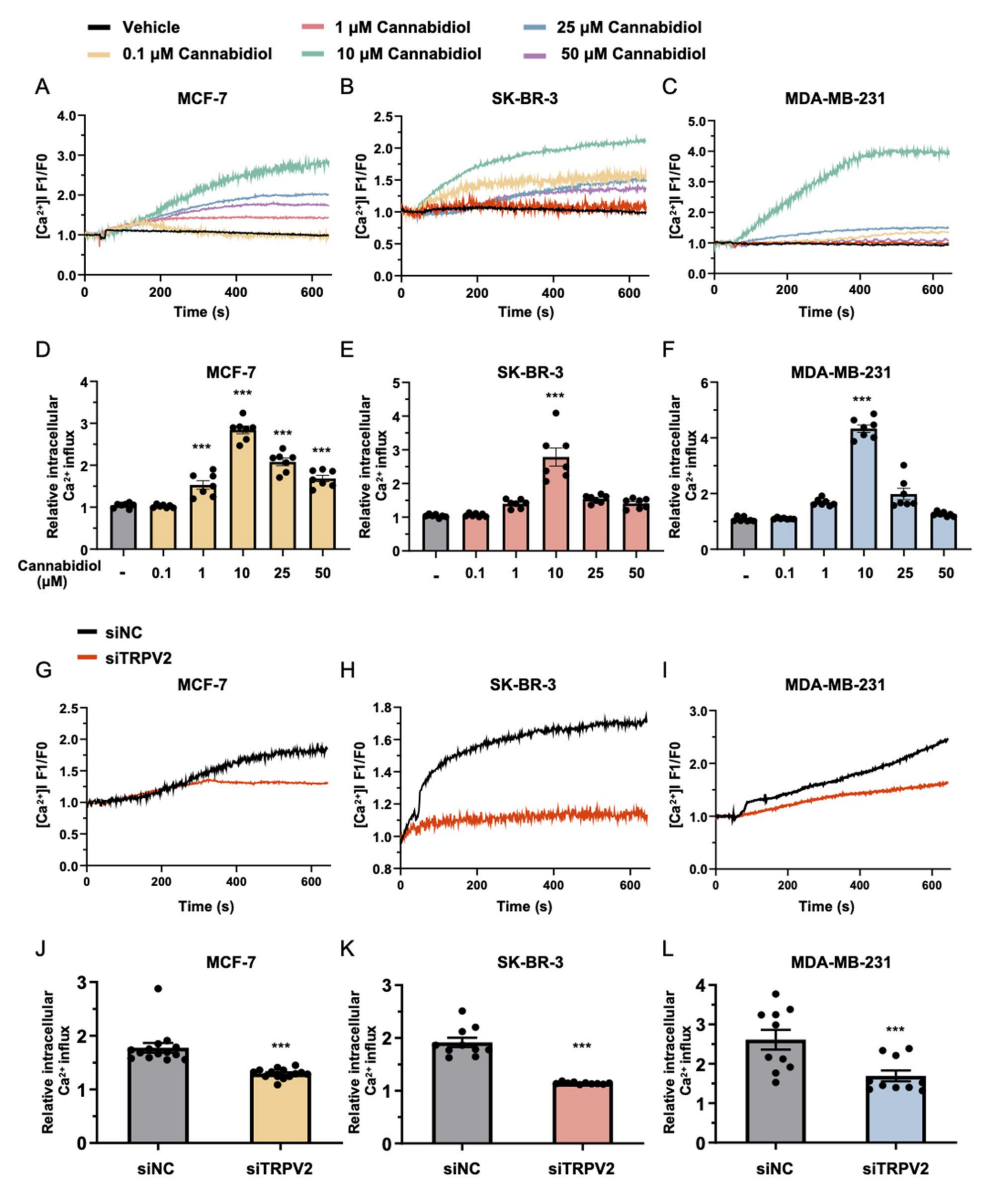
TRPV2 facilitates calcium entry in breast cancer cells. **(A-C)** Real-time changes of Fluo-4 labeled cytosolic Ca^2+^ fluorescence intensity in MCF-7 **(A)**, SK-BR-3 **(B)**, and MDA-MB-231 **(C)** cells upon stimulation with cannabidiol (*n* = 7). **(D-F)** Quantification of peak intracellular Ca^2+^ levels in MCF-7 **(D)**, SK-BR-3 **(E)**, and MDA-MB-231 **(F)** cells in response to cannabidiol stimulation (*n* = 7). **(G-I)** Dynamic changes of Fluo-4 labeled cytosolic Ca^2+^ fluorescence intensity upon 10 µM cannabidiol stimulation in MCF-7 **(G)**, SK-BR-3 **(H)**, and MDA-MB-231 **(I)** cells, comparing the effects of TRPV2 knockdown and control conditions (*n* = 10–14). **(J-L)** Assessment of peak intracellular Ca^2+^ levels in MCF-7 **(J)**, SK-BR-3 **(K)**, and MDA-MB-231 **(L)** cells with or without TRPV2 knockdown, following stimulation with 10 µM cannabidiol (*n* = 10–14). Error bar represents mean ± SEM. ***p < 0.001; Student’s t test in **(G-L)**, analysis of variance test (ANOVA) in **(A-F)**. NC, negative control

Recent studies have implicated the dysregulation of Ca^2+^ homeostasis as a contributing factor to cancer progression. Given the pivotal role of TRPV2 as a calcium-permeable cation channel and a crucial modulator of calcium handling [42, 46, 47], we then investigated the potential role of TRPV2 in mediating extracellular calcium influx in breast cancer. To accomplish this, we employed cannabidiol as a pharmacological tool to activate TRPV2 in breast cancer cells, and extra Fluo-4 labeled calcium buffer were applied to monitor and track the dynamic changes in calcium levels within the cellular context [48, 49]. Breast cancer cells were subjected to different concentrations of cannabidiol, and the data demonstrated a notable increase in Fluo-4 fluorescence intensity in MCF-7 (Fig. 3A and D), SK-BR-3 (Fig. 3B and E), and MDA-MB-231 (Fig. 3C and F) cell lines following treatment with 10 µM cannabidiol. This notable augmentation indicated an enhanced influx of calcium ions through the plasma membrane, triggered specifically by the presence of cannabidiol. Consequently, this influx led to an elevation in the intracellular calcium concentration within the breast cancer cells. The effect of TRPV2 knockdown on Ca^2+^ influx in response to TRPV2 channel activity stimulation by cannabidiol was further assessed in the aforementioned breast cancer cell lines. Our findings demonstrated that cannabidiol induced an increase in Ca^2+^ influx in the control group of breast cancer cells (Fig. 3G-H). However, this cannabidiol-induced elevation in extracellular Ca^2+^ influx was reversed in breast cancer cells with TRPV2 knockdown (Fig. 3G-H). These results together highlight the regulatory role of TRPV2 in the Ca^2+^ signaling pathway within breast cancer cells.

### Autophagy is activated in breast cancer

**Fig. 4:**
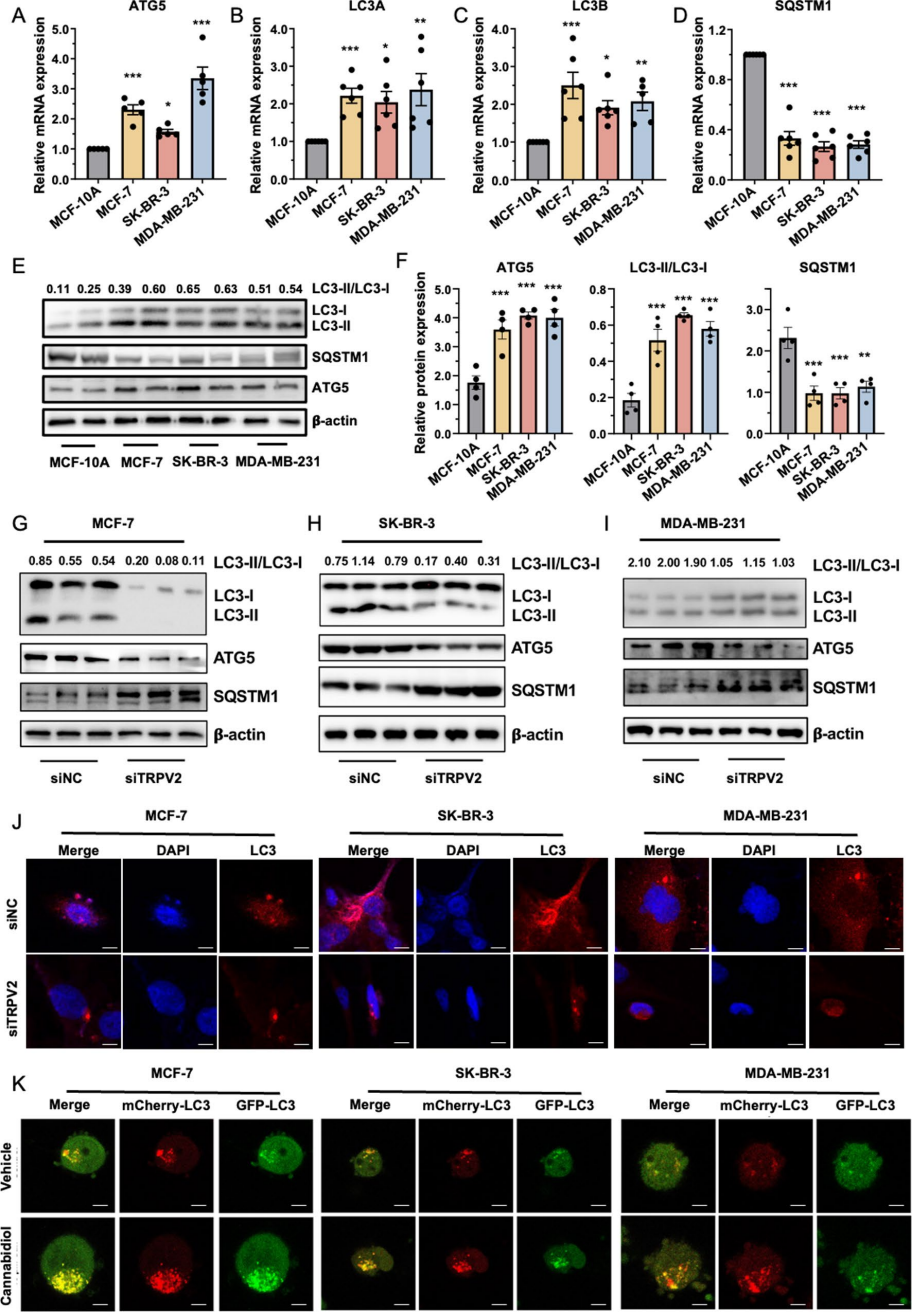
TRPV2 modulates autophagic activities in breast cancer cells. **(A-D)** Comparative analysis of relative mRNA expression levels of autophagy markers including ATG5 **(A)**, LC3A **(B)**, LC3B **(C)**, and SQSTM1 **(D)** in normal breast epithelial cells (MCF-10 A) and breast cancer cells (MCF-7, SK-BR-3, and MDA-MB-231) (*n* = 5–6). **(E-F)** Representative immunoblots **(E)** and quantification **(F)** of autophagic programs in normal breast epithelial cells (MCF-10 A) and breast cancer cells (MCF-7, SK-BR-3, and MDA-MB-231) (*n* = 4). **(G-I)** Representative immunoblots depicting the autophagic programs in breast cancer cells MCF-7 **(G)**, SK-BR-3 **(H)**, and MDA-MB-231 **(I)** with or without TRPV2 silencing (*n* = 6). **(J)** Representative image of GFP-LC3 puncta in breast cancer cells MCF-7 (left), SK-BR-3 (middle), and MDA-MB-231 (right) with or without TRPV2 knockdown (*n* = 10; scale bar, 30 μm). **(K)** Representative image of mCherry-LC3 and GFP-LC3 puncta in breast cancer cells MCF-7 (left), SK-BR-3 (middle), and MDA-MB-231 (right) under treatment of cannabidiol and the corresponding untreated control (*n* = 12; scale bar, 120 μm). Error bar represents mean ± SEM. *p < 0.05, **p < 0.01, ***p < 0.001; Analysis of variance test (ANOVA) in **(A-F)**. NC, negative control

Previous research has emphasized the significance of augmented autophagic flux as a tumor survival mechanism, aiding in the adaptation to metabolic and hypoxic stresses [50–52]. Experimental and structural studies have established the regulatory involvement of calcium signaling in the autophagy process [53–55]. Considering the prominent expression of TRPV2 in breast cancer (Fig. 1), we investigated whether this correlation is associated with elevated autophagic activity. Specifically, we evaluated the expression of key components involved in the autophagic program within normal breast epithelial cells (MCF-10 A) and breast cancer cells (MCF-7, SK-BR-3, and MDA-MB-231). A significant elevation was observed in both the mRNA levels and protein expression of ATG5, an essential factor for autophagic vesicle formation, in the three breast cancer cell lines compared to MCF-10 A (Fig. 4A and E-F). Moreover, our results demonstrated significantly increased mRNA expression of LC3A and LC3B, canonical markers for autophagosomes, in breast cancer cells compared to normal breast epithelial cells (Fig. 4B). As the process of autophagosome formation involves the conjugation of LC3 to phosphatidylethanolamine, leading to the transformation from LC3-I to LC3-II, we subsequently evaluated the levels of both LC3-I and LC3-II to determine the activity of autophagy in breast cancer. Western blot analysis revealed a noticeable increase in the ratio of LC3-II/LC3-I in MCF-7, SK-BR-3, and MDA-MB-231 cells (Fig. 4B-C and E-F), indicating the conversion of more LC3-I to LC3-II in breast cancer cells. Conversely, autophagic substrate SQSTM1, which is degraded efficiently following the incorporation into autophagosome [56], was remarkably downregulated in cancer cells (Fig. 4D-F). These findings collectively suggest elevated autophagic activity in breast cancer.

### TRPV2 modulates autophagic flux in breast cancer

We next investigated regulatory role of TRPV2 on autophagy in breast cancer. Quantitative PCR analysis demonstrated a significant reduction in the expression levels of ATG, LC3A, and LC3B, along with an increase in SQSTM1, following TRPV2 knockdown in MCF-7, SK-BR-3, and MDA-MB-231 breast cancer cells (Figure S4A-C). Consistently, western blot analysis further revealed decreased expression of ATG, a reduced ratio of LC3-II to LC3-I, and increased SQSTM1 in breast cancer cells with TRPV2 knockdown (Fig. 4G-I). To identify autophagosome/autolysosome accumulation, we employed GFP-LC3 dots as an indicator of LC3-II molecules associated with nascent phagophores and autophagosomal vesicles [57, 58]. Our data indicated a decrease in LC3 dots in TRPV2-silenced MCF-7, SK-BR-3, and MDA-MB-231 breast cancer cells (Fig. 4J and Figure S4 D-F). These findings collectively suggest that silencing TRPV2 suppresses autophagic activities in breast cancer cells.

In contrast, the application of the TRPV2 agonist cannabidiol to MCF-7, SK-BR-3, and MDA-MB-231 breast cancer cells resulted in increased expression of ATG, LC3A, and LC3B, along with reduced SQSTM1, as observed in our quantitative PCR analysis (Figure S5A-C). Likewise, western blot analysis revealed increased expression of ATG, an elevated ratio of LC3-II to LC3-I, and decreased SQSTM1 in breast cancer cells upon TRPV2 activation by cannabidiol (Figure S5 D-F). Additionally, an augmented number of GFP-LC3 dots, indicating increased autophagosome/autolysosome accumulation, were observed in breast cancer cells following cannabidiol treatment (Figure S5G-I). Furthermore, we examined the expression of tandem fluorescent-tagged LC3 (mCherry-GFP-LC3) to monitor the fusion of autophagosomes with lysosomes, thereby assessing autophagosome maturation. In this particular experimental assay, the lysosomal environment causes quenching of the GFP fluorescent signal, while mCherry fluorescence remains relatively stable within acidic compartments [59, 60]. Consequently, autophagosomes and amphisomes were labeled with a yellow fluorescence signal, whereas red fluorescence signals were observed in autolysosomes. Remarkably, we observed an increased number of yellow (mCherry^+^GFP^+^) puncta in breast cancer cells after cannabidiol treatment compared to control cells (Fig. 4K and Figure S5J-L), indicating enhanced autolysosome-lysosome fusion during the autophagy process. Taken together, these data suggest that TRPV2 activation promotes autophagic flux.

### TRPV2-mediated breast cancer progression is dependent on autophagy

**Fig. 5:**
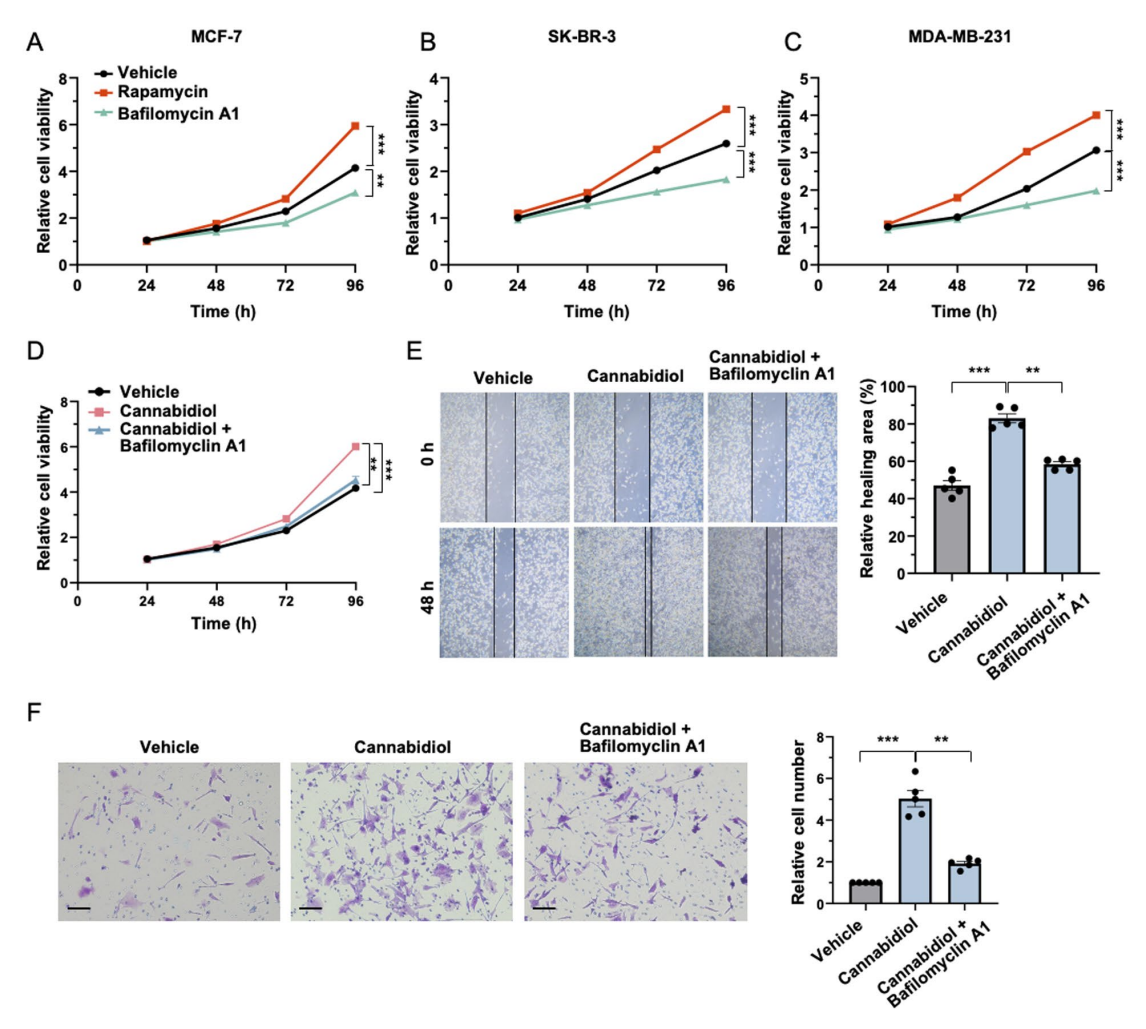
TRPV2 facilitates breast cancer progression via activating autophagy. **(A-C)** Quantitative analysis of cell viability in MCF-7 **(A)**, SK-BR-3 **(B)**, and MDA-MB-231 **(C)** breast cancer cell lines. Cells were either left untreated or treated with bafilomycin A1 or rapamycin. (*n* = 7–8). **(D)** Quantification of cell viability in breast cancer cells. The cells were either left untreated, or treated with TRPV2 activator cannabidiol in the absence or presence of the autophagy blocker bafilomycin A1 (*n* = 6). **(E)** Representative image (left) and quantification (right) demonstrating invaded cells by transwell assay. The cells were subjected to different conditions, including the untreated group, treatment with the TRPV2 activator cannabidiol, and treatment with cannabidiol along with the autophagy blocker bafilomycin A1 (*n* = 5). **(F)** Representative image (left) and quantitative analysis (right) depicting the wound healing area in breast cancer cells. The cells were exposed to various conditions, including the untreated group, treatment with the TRPV2 activator cannabidiol, and treatment with cannabidiol in combination with the autophagy blocker bafilomycin A1(*n* = 5; scale bar, 15 μm). Error bar represents mean ± SEM. **p < 0.01, ***p < 0.001; Analysis of variance test (ANOVA) in **(A-F)**

We next investigated the role of autophagy in breast cancer by pharmacologically modulating autophagic activity. In this study, we utilized bafilomycin A1, a macrolide antibiotic renowned for its capacity to hinder the fusion process between autophagosomes and lysosomes, thereby impeding the later stages of autophagy. Additionally, we employed rapamycin, a widely recognized inducer of autophagy that has demonstrated effectiveness across diverse cell types [61]. breast cancer cells were treated with either bafilomycin A1 or rapamycin, and subsequently, cell viability was assessed. The results revealed that breast cancer cells treated with rapamycin exhibited elevated proliferation, as evidenced by a notable increase in cell viability (Fig. 5A-C). Conversely, treatment with bafilomycin A1 led to a decrease in the proliferation of breast cancer cells, indicating the suppression of cell growth (Fig. 5A-C). These findings underscore that heightened autophagic activity contributes to the progression of breast cancer.

In light of the demonstrated modulatory role of TRPV2 in breast cancer (Fig. 4), we proceeded to investigate the potential dependency of TRPV2-mediated regulatory effects on autophagy. To this end, breast cancer cells were treated with the TRPV2 activator cannabidiol, in the absence or presence of the autophagy blocker bafilomycin A1. Notably, the results of the MTT assay revealed that the proliferative effect induced by cannabidiol-mediated TRPV2 activation was repressed in the presence of the autophagy inhibitor bafilomycin A1 (Fig. 5C). Similarly, the promotion of cancer cell invasion by cannabidiol, as observed in the transwell assay, was significantly diminished when autophagy was inhibited (Fig. 5D). Furthermore, wound healing assay results demonstrated a significant increase in the healing area of the breast cancer cells following cannabidiol treatment. However, this effect was reversed upon the presence of the autophagy blocker bafilomycin A1 (Fig. 5E). Collectively, these findings suggest that TRPV2 promotes the progression of breast cancer by upregulating autophagy.

### TRPV2 regulates autophagy through CaMKKβ-AMPK-ULK1 signaling cascade

**Fig. 6:**
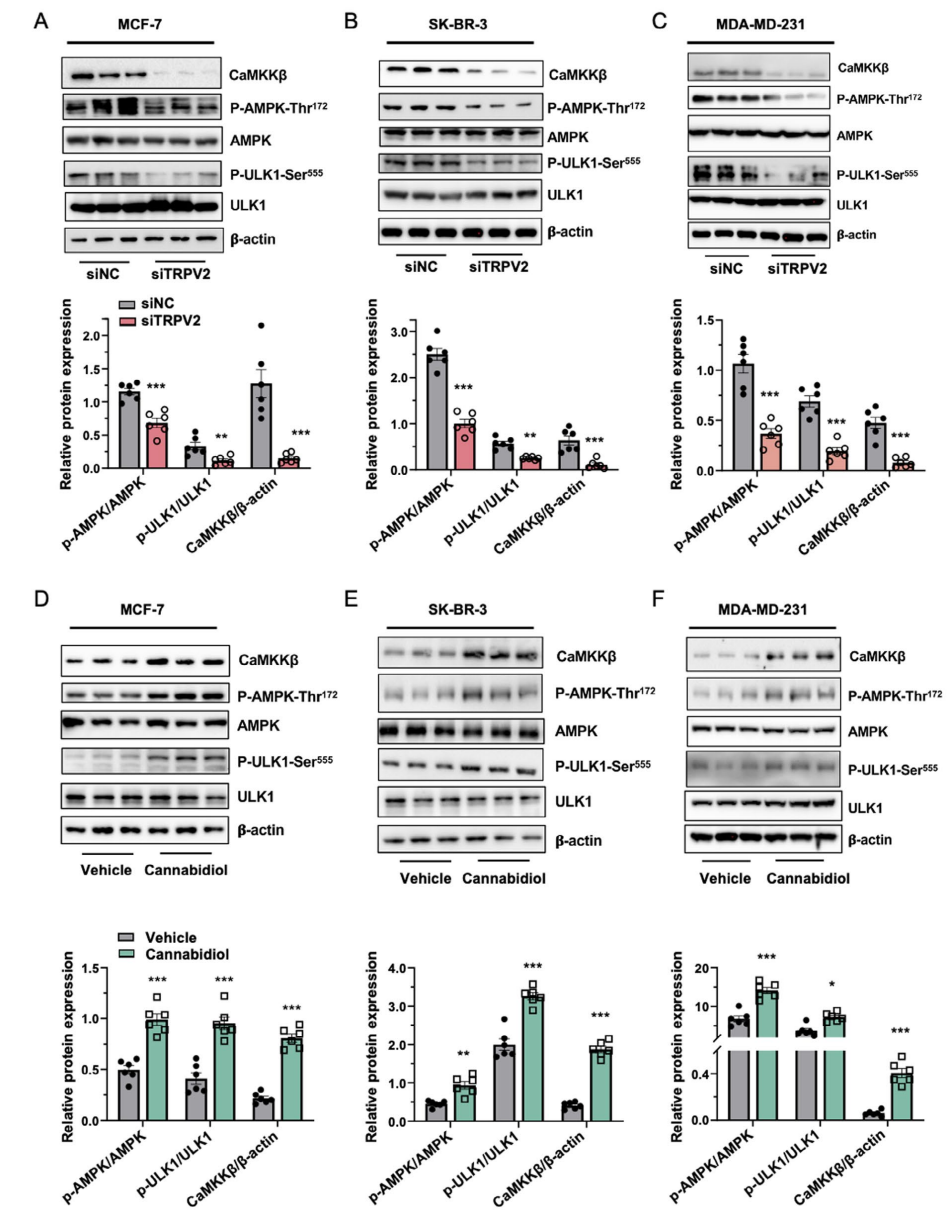
TRPV2 controls autophagy in breast cancer via the CaMKKβ-AMPK-ULK1 signaling cascade. **(A-C)** Representative immunoblots (upper panel) and quantification (lower panel) demonstrating the expression levels of AMPK, ULK1, and CaMKKβ, in breast cancer cell lines MCF-7 **(A)**, SK-BR-3 **(B)**, and MDA-MB-231 **(C)** with or without TRPV2 knockdown (*n* = 6). **(D-F)** Representative immunoblots (upper panel) and quantitative analysis (lower panel) of the expression levels of AMPK, ULK1, and CaMKKβ, in breast cancer cell lines MCF-7 **(D)**, SK-BR-3 **(E)**, and MDA-MB-231 **(F)** treated with or without TRPV2 activator cannabidiol (*n* = 6). Error bar represents mean ± SEM. **p < 0.01, ***p < 0.001; Student’s t test in **(A-F)**. NC, negative control

To elucidate the mechanistic connection between the TRPV2 calcium channel and autophagy, we investigated the molecular targets activated by calcium ions in the autophagic cascade. Given the implications of Ca^2+^/calmodulin-dependent kinase β (CaMKKβ) in autophagy regulation [62], coupled with its reported modulation by TRP channels [63], we sought to determine the potential involvement of CaMKKβ in mediating TRPV2-induced autophagy in the context of breast cancer. Our findings revealed a significant reduction of CaMKKβ in breast cancer cells upon TRPV2 silencing (Fig. 6A-C), while TRPV2 activation with cannabidiol treatment led to increased phosphorylation of CaMKKβ (Fig. 6D-E). In light of the well-established role of AMP-activated protein kinase (AMPK) as a downstream effector of CaMKKβ and its essential function as a canonical initiator of the autophagic cascade, we further assessed the expression of AMPK in our experimental setting. We observed a decrease in phosphorylated AMPK at threonine 172 in breast cancer cells with TRPV2 knockdown (Fig. 6A-C), while treatment with cannabidiol resulted in increased phosphorylation of AMPK at threonine 172 (Fig. 6D-E). Previous studies have shown that AMPK stimulates autophagy by phosphorylating ULK1 at serine 555. Subsequently, ubiquitination of activated ULK1 by the E3 ligase NEDD4L leads to proteasomal degradation of ULK1, thus limiting autophagic flux following an initial autophagy response. Consistent with our observations for AMPK expression, knockdown of TRPV2 in breast cancer cells resulted in inhibition of ULK1 phosphorylation at serine 555 (Fig. 6A-C), while cannabidiol treatment led to upregulation of ULK1 phosphorylation (Fig. 6D-E). Taken together, these findings provide evidence that TRPV2 modulates autophagy in breast cancer through the CaMKKβ-AMPK-ULK1 cascade.

### TRPV2 promotes growth of xenograft breast tumors through activating autophagy

**Fig. 7:**
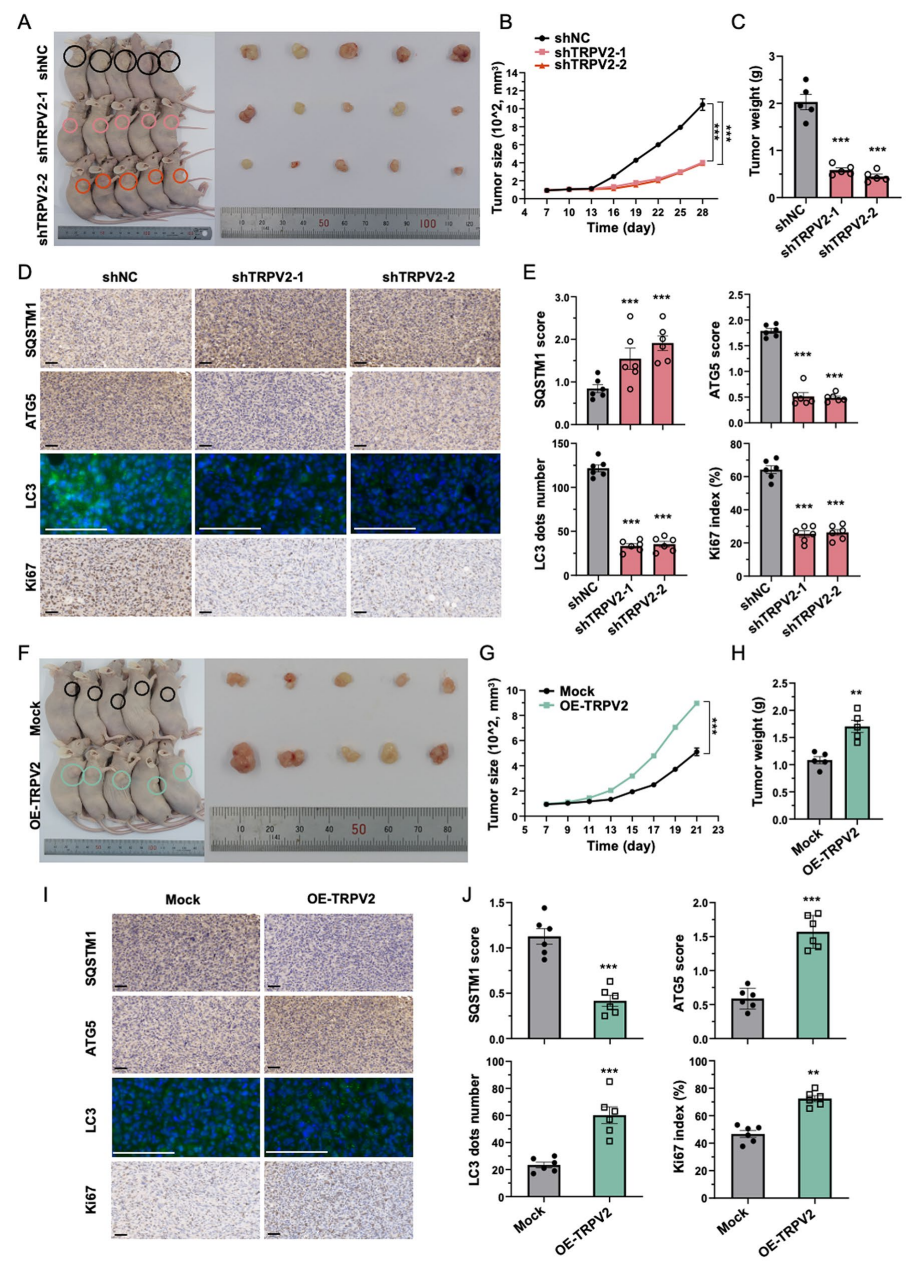
TRPV2 facilitates the growth of xenograft breast tumors via modulating autophagy. **(A)** Images showing the animals prior to euthanasia and the collected tumors from each annotated group at the experimental endpoint. The presence of xenograft breast tumors is indicated by circles (*n* = 5). **(B)** Measurement of tumor size derived from xenograft tumors in the fourth left mammary fat pad of Balb/c nude mice during the experimental period (*n* = 5). **(C)** Quantification of the weight of xenograft tumors derived from MDA-MB-231 cells with or without TRPV2 knockdown at the experimental endpoint (*n* = 5). **(D-E)** Representative images **(D)** and corresponding quantification **(E)** of IHC staining of SQSTM1, ATG5, LC3 and Ki67 in tumor sections from the control group and TRPV2 knockdown group (*n* = 6; scale bar, 50 μm). **(F)** Images of the mice prior to euthanasia, along with the corresponding collected tumors from each annotated experimental group at the endpoint of the study. Circular annotations highlight the presence of xenograft breast tumors. (*n* = 5). **(G)** The size of xenograft tumors originating from the fourth left mammary fat pad of Balb/c nude mice was measured in both the control group and the TRPV2 overexpression group. The measurements were conducted over the experimental period (*n* = 5). **(H)** Measurement of the weight of xenograft tumors derived from MDA-MB-231 cells with or without TRPV2 overexpression at the experimental endpoint (*n* = 5). **(I-J)** Representative images **(I)** and corresponding quantification **(J)** of IHC staining for SQSTM1, ATG5, LC3, and Ki67 in tumor sections obtained from both the control group and the TRPV2 overexpression group (*n* = 6; scale bar, 50 μm). Error bar represents mean ± SEM. **p < 0.01, ***p < 0.001; Student’s t test in **(F-J)**, analysis of variance test (ANOVA) in **(A-E)**. NC, negative control; OE, overexpression

Considering the aggressive nature of triple negative breast cancer (TNBC) and its limited therapeutic options, we proceeded to elucidate the tumorigenic effect of TRPV2 in vivo by injecting the TNBC cell line MDA-MB-231 cells with control vector, shTRPV2-1, or shTRPV2-2 vector into Balb/c nude mice. The knockdown of TRPV2 in cancer cells resulted in a significant reduction in both tumor size and weight, indicating a suppressed tumor growth (Fig. 7A-C). Consistently, we observed a substantial decrease in Ki67 (a marker of dividing cells) levels in tumor section from TRPV2 silencing group (Fig. 7D-E). Moreover, the knockdown of TRPV2 in xenograft breast tumors led to the suppression of autophagy, as evidenced by evaluated SQSTM1 levels and decreased expression of ATG5 and LC3 (Fig. 7D-E).

To corroborate our findings using a gain-of-function approach, we further introduced stable overexpression of the TRPV2 channel in MDA-MB-231 breast cancer cells (OE TRPV2) in xenograft tumor model. Tumors derived from MDA-MB-231 cells with stable TRPV2 overexpression exhibited accelerated growth compared to the control group (Mock), as indicated by increased tumor size and weight (Fig. 7F-H). This pro-tumorigenic effect of TRPV2 overexpression was further verified by the elevated expression of Ki67 in the tumor sections (Fig. 7I-J). Additionally, we noted a decline in SQSTM1 levels and an upregulation of ATG5 and LC3 expression (Fig. 7I-J), indicative of enhanced autophagic activity following TRPV2 overexpression.

Overall, these data indicate that TRPV2 plays a role in promoting the growth of xenograft breast tumors through the activation of autophagy.

## Discussion

The intricate interplay of Ca^2+^ homeostasis during lactation and its pervasive involvement in tumorigenesis suggests a potential contributory role in the development of breast cancer [64]. Specifically, Ca^2+^ plays a crucial role in regulating breast cancer growth by interacting with calmodulin and influencing cell cycle progression [65]. Additionally, Ca^2+^ entry activates MAP kinase (ERK1/2) in MCF-7 cells, leading to breast cancer proliferation [30]. TRPV2, a nonselective cation channel with high Ca^2+^ permeability, exhibits variable expression across different tissue and cell types [66]. The expression of TRPV2 varies in different tissues and cell types. Notably, TRPV2 expression is prominent in the brain, lung, and spleen [67]. In addition to normal tissues, TRPV2 is expressed in various tumor cells, including gliomas, glioblastoma, and hepatoma cells [68–70]. In myeloma cancer, elevated TRPV2 expression correlates with bone tissue damage and poor prognosis [71]. Likewise, in prostate cancer, TRPV2 is overexpressed and linked to the castration-resistant phenotype and metastasis [72]. In our study, we observed higher TRPV2 expression in breast cancer cell lines (MCF-7, SK-BR-3, and MDA-MB-231) compared to normal breast cells (MCF-10 A). This observation aligns with our analysis of clinical samples from breast cancer patients, indicating elevated TRPV2 expression in tumor areas compared to adjacent non-tumor tissue. Moreover, in MDA-MB-231 cells with higher metastatic ability, we found increased TRPV2 expression compared to MCF-7 cells with lower metastatic ability. This observation is consistent with a previous study that found higher TRPV2 expression in a poorly differentiated bladder cancer cell line with greater metastatic potential compared to a well-differentiated cancer cell line with lower metastatic ability [73]. Additionally, in contrast to previous studies reporting a positive correlation between TRPV2 expression and high risk of relapse in triple-negative breast cancer [74], our study demonstrated higher TRPV2 expression specifically in more advanced tumor stages, particularly stage 3, which has not been previously reported. These findings suggest that TRPV2 may play a vital role in the progression of breast cancer, underscoring its significant contribution to the advancement of the disease. Furthermore, these results further solidify TRPV2 as a promising candidate for a biomarker that could enhance the accuracy of breast cancer staging.

TRPV2 predominantly localizes within intracellular compartments, such as the Endoplasmic Reticulum, in unstimulated cells, where it potentially functions as an endosomal calcium release channel involved in endosome fusion and/or exocytosis [46]. Various stimuli, including heat (> 52 °C), ligands like cannabidiol, 2-APB, probenecid, and mechanical stresses, can activate TRPV2 [48, 75–78]. In our study, we utilized cannabidiol, a TRPV2 activator, to treat breast cancer cells. We observed that cannabidiol induced an increase in the influx of Ca^2+^ across the cell membrane. Furthermore, the activation of TRPV2 by cannabidiol was found to enhance the proliferative and metastatic potential of breast cancer cells. To further elucidate the role of TRPV2 in breast cancer, we utilized a knockdown strategy both in vitro and in vivo. Our investigation revealed that silencing TRPV2 significantly attenuated malignant characteristics, including proliferation, migration, and invasion, in vitro. Notably, this effect was also observed in vivo, where tumorigenesis was markedly inhibited. Mechanistically, while prior research has shed light on the capacity of cannabidiol to induce apoptosis in glioblastoma cancer cells by triggering Ca^2+^ overload [69], building upon these findings, our study provides pioneering evidence of the involvement of TRPV2 in promoting cancer progression in breast cancer through the activation of autophagy. This activation confers a survival advantage to cancer cells. Our study unveils a novel mechanism that contributes to the pathogenesis of breast cancer, further advancing our understanding of this complex disease.

A previous study demonstrated that TRPM3, another TRP channel, increases Ca^2+^ influx and promotes clear cell renal cell carcinoma growth by stimulating autophagy [79]. However, the precise mechanism by which TRPV2 regulates autophagy through Ca^2+^ modulation in breast cancer cells remains unknown. CaMKKβ plays a crucial role in the initial formation of autophagosomes through Ca^2+^ signaling [55, 80]. Specifically, Ca^2+^ interacts with CaM, an intracellular receptor, to regulate various cellular functions [81, 82]. Upon Ca^2+^ binding, the affinity of CaM for other CaM kinases, including CaMKKβ, is enhanced, triggering the Ca^2+^/CaM cascade [83]. In our study, we unveil a novel mechanism by which TRPV2 controls autophagy in breast cancer cells. Our findings demonstrate that the TRPV2-CaMKKβ-AMPK signaling pathway cascade induces autophagy by phosphorylating ULK1 at serine 555. This phosphorylation event activates the ULK complex, initiating autophagosome formation and autophagic flux, thereby modulating breast cancer cell progression. Our elucidation of this TRPV2-mediated regulation of autophagy in breast cancer cells provides valuable insights for the development of targeted therapies aimed at manipulating autophagy pathways to control breast cancer progression.

The mechanisms underlying the translocation of TRPV2 from the ER to the cell membrane in breast cancer cells remain elusive. TRPV2 localization is modulated by various stressful conditions. For instance, in the presence of serum, TRPV2 relocates from the ER membrane to the plasma membrane, where it interacts with cellular components [67]. Conversely, upon serum removal, a significant proportion of TRPV2 returns to the ER membrane. Notably, IGF-I, EGF, and PDGF are key active components present in serum that have been reported to induce TRPV2 translocation [67, 84]. Among the intracellular signals triggered by IGF-I, PI3K emerges as a crucial mediator. Notably, studies have demonstrated that the addition of LY294002, a specific PI3K inhibitor, markedly suppresses TRPV2 translocation, highlighting the vital role of PI3K in facilitating the translocation of TRPV2 from the ER membrane to the plasma membrane [67]. Moreover, other studies have revealed upregulated expression of PI3K in breast cancer cells, further implying its involvement in trafficking of TRPV2 to the plasma membrane [85–88]. Therefore, further investigations are warranted to confirm the dependence of the translocation process of TRPV2 in breast cancer cells on PI3K. These investigations hold the potential to uncover novel therapeutic targets for upstream interventions aimed at modulating TRPV2-mediated functions in breast cancer.

## Conclusions

In conclusion, our study highlights the high expression of TRPV2, a calcium-permeable TRP channel, in breast cancer, highlighting its potential utility as a biomarker for assessing the malignancy stage. Our findings demonstrate that TRPV2 actively promotes breast cancer progression by activating autophagy, offering a novel avenue for the development of therapeutic interventions in breast cancer treatment by specifically targeting TRPV2.

## Electronic supplementary material

Below is the link to the electronic supplementary material.

Supplementary Material 1

## Data Availability

No datasets were generated or analysed during the current study.
