# Supplementary material for: TRPV2 calcium channel promotes breast cancer progression potential by activating autophagy
Source: Cancer Cell Int. 2024 Sep 27;24:324. doi: 10.1186/s12935-024-03506-y (PMC11438410; doi:10.1186/s12935-024-03506-y)
Supplement: Supplementary file 1 — Supplementary Material 1 [file 12935_2024_3506_MOESM1_ESM.pdf]

Supplementary material

Figure S1

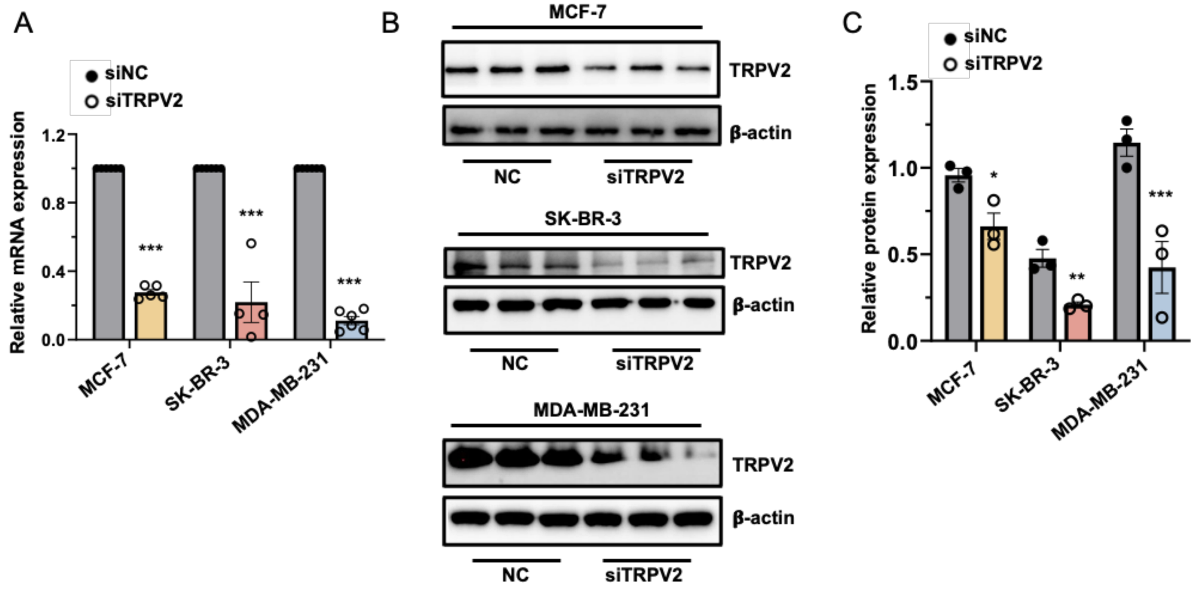

Figure S1: TRPV2 expression is decreased in breast cancer cell lines upon the application of TRPV2 siRNA.

(A) Transcript expression of TRPV2 in breast cancer cells treated with si-NC or si-TRPV2 (n = 4-6).

(B) Representative immunoblots (left) and quantification (right) of TRPV2 protein expression in breast cancer cell following si-NC or si-TRPV2 treatment (n = 3).

Error bar represents mean ± SEM. *$p < 0.05$, **$p < 0.01$, ***$p < 0.001$; Student's t test in (A-C).

NC, negative control.

Figure S2

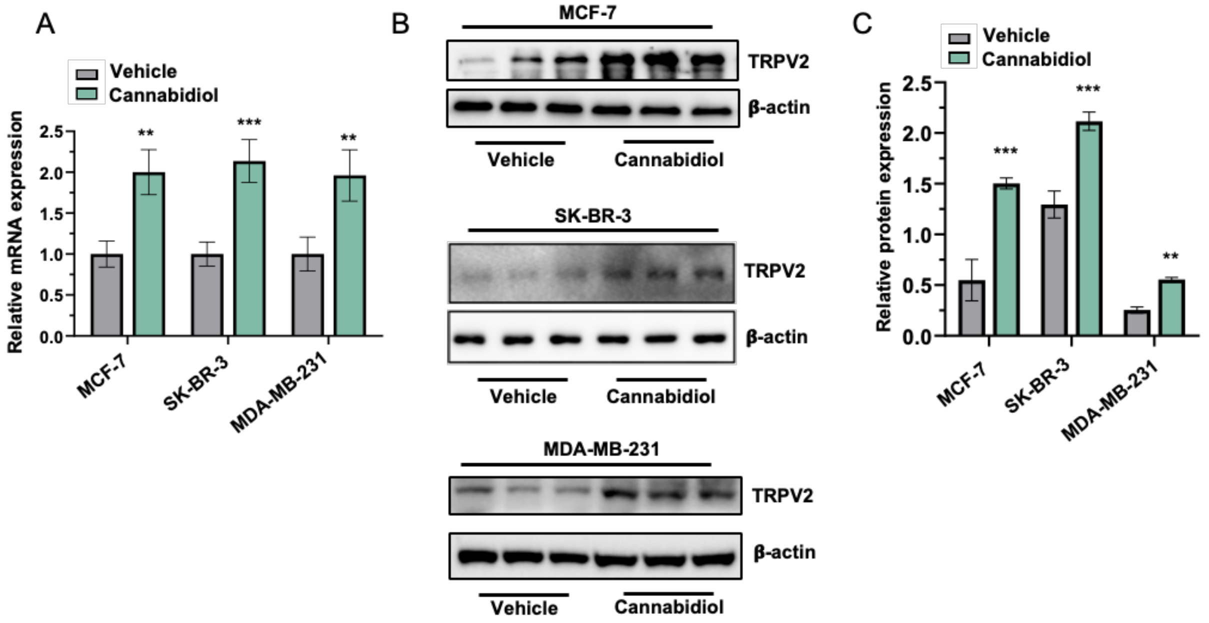

Figure S2: Application of cannabidiol increases the expression of TRPV2 in breast cancer cell lines.

(A) Expression of TRPV2 transcript in breast cancer cells treated with or without cannabidiol treatment (n = 6).

(B-C) Representative immunoblots (B) and translational analysis (C) of TRPV2 expression in breast cancer cell treated with or without cannabidiol (n = 3).

Error bar represents mean ± SEM. **$p < 0.01$, ***$p < 0.001$; Student's t test in (A-C).

Figure S3

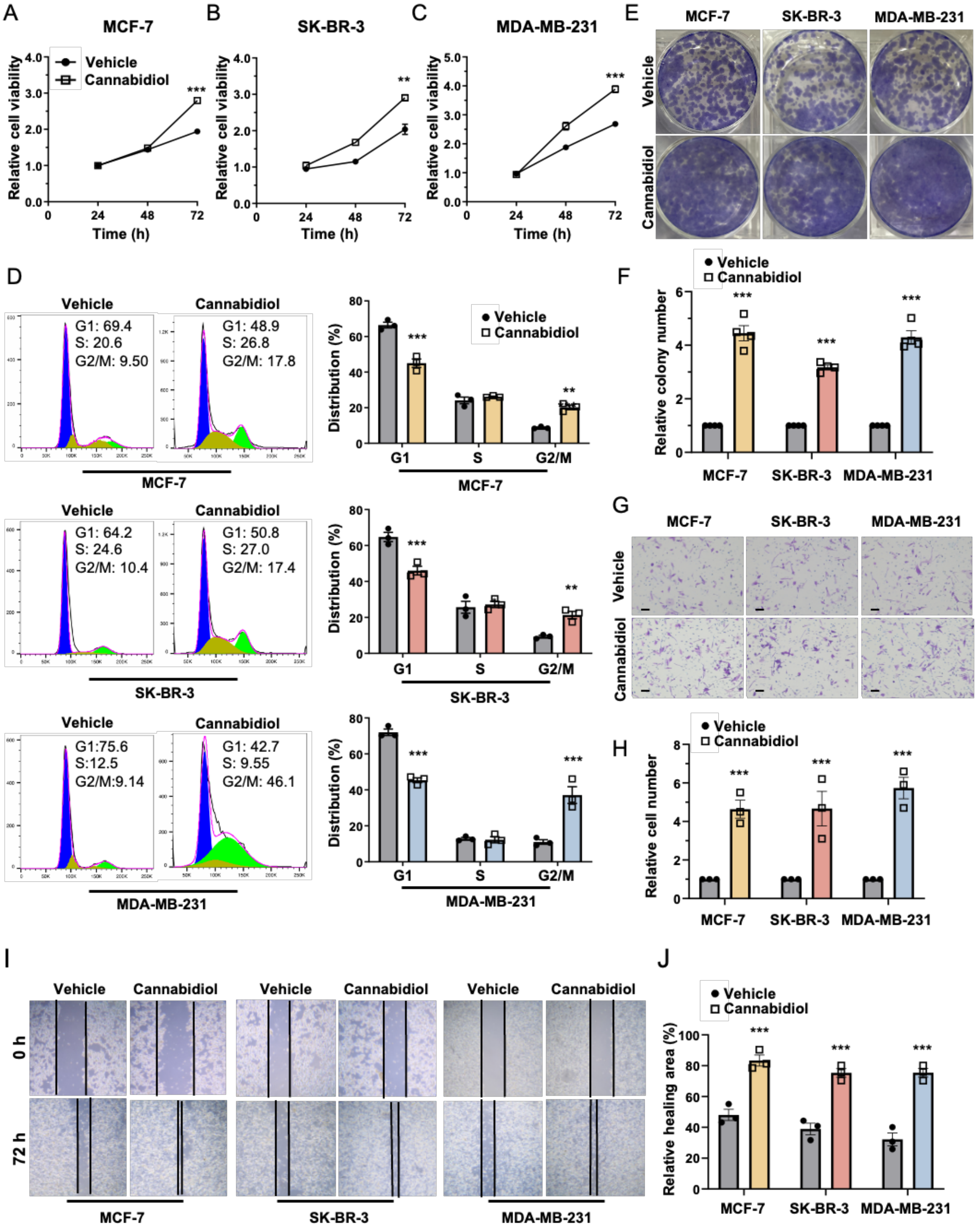
A
MCF-7
Vehicle
Cannabidiol
Relative cell viability
Time (h)
B
SK-BR-3
C
MDA-MB-231
E
MCF-7
SK-BR-3
MDA-MB-231
Vehicle
Cannabidiol
D
Vehicle
Cannabidiol
G1: 69.4
S: 20.6
G2/M: 9.50
G1: 48.9
S: 26.8
G2/M: 17.8
MCF-7
Distribution (%)
G1
S
G2/M
G1: 64.2
S: 24.6
G2/M: 10.4
G1: 50.8
S: 27.0
G2/M: 17.4
SK-BR-3
G1:75.6
S:12.5
G2/M:9.14
G1: 42.7
S: 9.55
G2/M: 46.1
MDA-MB-231
F
Relative colony number
G
H
Relative cell number
I
0 h
72 h
J
Relative healing area (%)

Figure S3: Activation of TRPV2 by cannabidiol promotes the proliferation and metastasis of breast cancer.

(A-C) Cell viability quantification of MCF-7 (A), SK-BR-3 (B), and MDA-MB-231(C) breast cancer cells with or without activation of TRPV2 by cannabidiol, assessed using the MTT assay (n = 6).

(D) Representative flow plot (left) and quantitative analysis (right) illustrating the cell cycle distribution in MCF-7, SK-BR-3, and MDA-MB-231 cells with or without cannabidiol treatment for TRPV2 activation (n = 3).

(E-F) Representative colony formed image (E) and quantitative analysis (F) in breast cancer cells with or without TRPV2 activation by cannabidiol application (n = 4).

(G-H) Representative image (G) and quantification (H) of invaded cells in breast cancer cells treated with or without cannabidiol by transwell assay (n = 3; scale bar, 10 μm).

(I-J) Representative image (I) and quantitative analysis (J) of the wound healing area in breast cancer cells with or without cannabidiol treatment (n = 3).

Error bar represents mean ± SEM. $^{**}p < 0.01$, $^{***}p < 0.001$; Student's t test in (E-J), analysis of variance test (ANOVA) in (A-D).

Figure S4

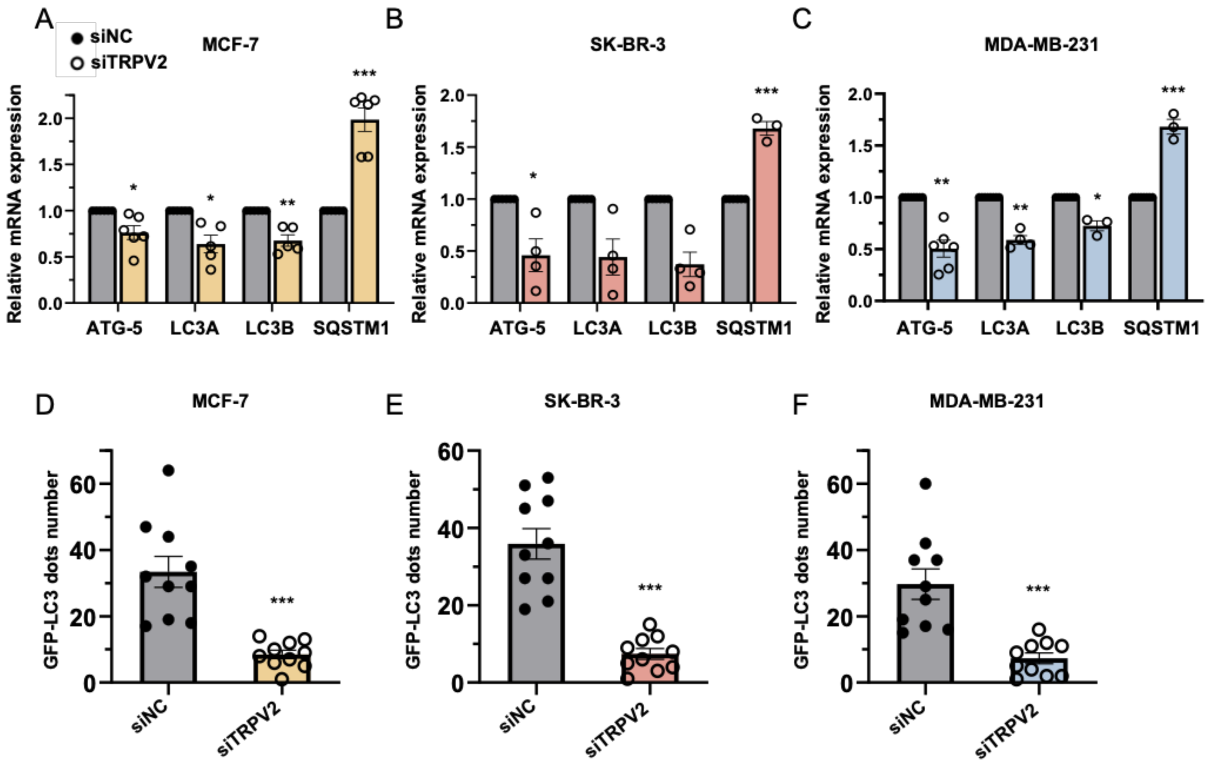

Figure S4: Silencing TRPV2 compromises autophagic activities in breast cancer.

(A-C) Relative mRNA expression of autophagic programs in breast cancer cells MCF-7 (A), SK-BR-3 (B), and MDA-MB-231 (C) with or without TRPV2 knockdown (n = 3-6).

(D-F) Quantification of GFP-LC3 dots in breast cancer cells MCF-7 (D), SK-BR-3 (E), and MDA-MB-231 (F) with or without TRPV2 silencing (n = 10).

Error bar represents mean ± SEM. *p < 0.05, **p < 0.01, ***p < 0.00; Student's t test in (A-F).

NC, negative control.

Figure S5

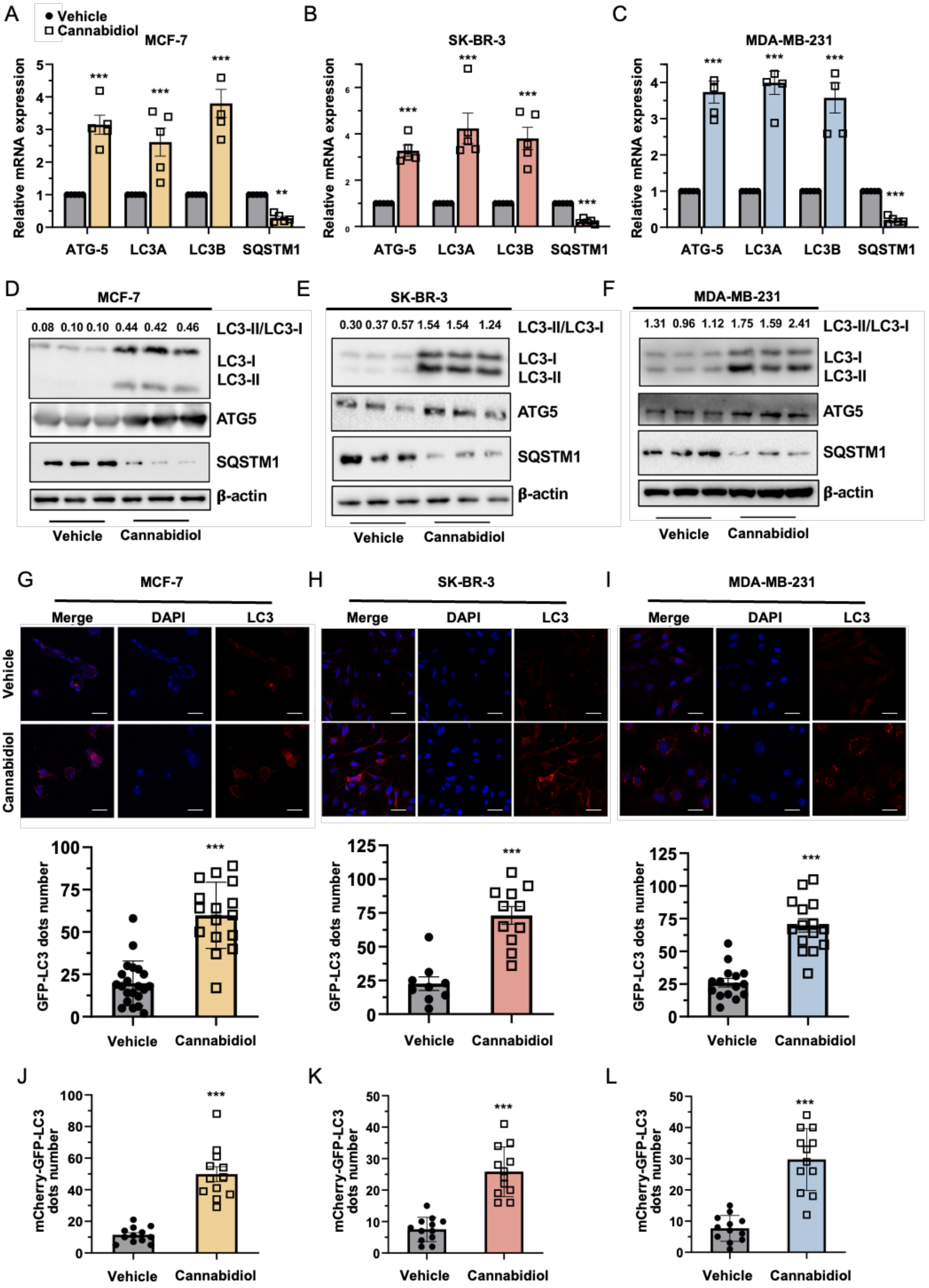
A
Vehicle
Cannabidiol
MCF-7
Relative mRNA expression
ATG-5
LC3A
LC3B
SQSTM1
B
SK-BR-3
C
MDA-MB-231
D
MCF-7
0.08 0.10 0.10 0.44 0.42 0.46
LC3-II/LC3-I
LC3-I
LC3-II
ATG5
SQSTM1
β-actin
Vehicle
Cannabidiol
E
SK-BR-3
0.30 0.37 0.57 1.54 1.54 1.24
F
MDA-MB-231
1.31 0.96 1.12 1.75 1.59 2.41
G
H
I
Merge
DAPI
LC3
GFP-LC3 dots number
J
K
L
mCherry-GFP-LC3 dots number

Figure S5: Activating TRPV2 promotes autophagic activities in breast cancer.

(A-C) Relative mRNA levels of autophagic programs in breast cancer cells MCF-7 (A), SK-BR-3 (B), and MDA-MB-231 (C) treated with or without cannabidiol (n = 5).

(D-F) Representative immunoblots of autophagic programs in breast cancer cells MCF-7 (D), SK-BR-3 (E), and MDA-MB-231 (F) with or without cannabidiol treatment (n = 6).

(G-I) Representative image (upper panel) and quantitative analysis (lower panel) of GFP-LC3 puncta in breast cancer cells MCF-7 (G) (n = 16-21), SK-BR-3 (H) (n = 9-11), and MDA-MB-231 (n = 15) (I) under treatment of cannabidiol and the untreated control (n = 9-16; scale bar, 30 μm).

(J-L) Quantification of mCherry-LC3 and GFP-LC3 dots in breast cancer cells MCF-7 (J), SK-BR-3 (K), and MDA-MB-231 (L) treated cannabidiol or left untreated (n = 12).

Error bar represents mean ± SEM. ***$p < 0.001$; Student's t test in (A-C and G-L).

Table S1: List of primers.

**Oligonucleotides**		
Gene	Forward sequence	Reverse sequence
GAPDH	GTCTCCTCTGACTTCAACAGCG	ACCACCCTGTTGCTGTAGCCAA
TRPA1	TGTGACGATATGGACACCTTCT	TTGAAGTTTCGGAGATTTGGGTT
TRPC1	GTAAATGGATTTGCTCGCAT	TGGTTAATTTCTTGGATAAA
TRPC3	AGCCGAGCCCCTGGAAAGACAC	CCGATGGCGAGGAATGGAAGAC
TRPC4	AATTACTCGTCAACAGGCGGC	CACCACCACCTTCTCCGACTT
TRPC5	AAGTTTCGAATTGAGGAGCAGATG	AATCTCTGATGGCATCGCACA
TRPC6	GCCTCATGATTATTTCTGCAAGTGTAC	TGAACTCTTTCTCAATGTTGGCAA
TRPC7	GTCCGAATGCAAGGAGATCT	TGGGTTGTACTTTGCACCTC
TRPV1	CAGGCTCTATGATCGCAGGAG	TTTGAACTCGTTGTCTGTGAGG
TRPV2	CATCTTCACCGCTGTTGCCTAC	CCTAGCAGGATAAGGATGTGGC
TRPV3	GGTAACTGTGATGACATGGACTC	ACCAGCAACTCTACCAACTCC
TRPV4	GATGGGCGACCAAATCTGC	GAGGACTCATATAGGGTGGACTC
TRPV5	GGTCATCTTGGGATTTGCCTCC	CAAGTCCACGTCGTAGTTGGCA
TRPV6	ACTGACCTCGACTCTCTATGAC	GTGGTGATGATAAGTTCCAGCAG
TRPM1	CCACCCGAGGGAGTCAGCAG	TGGCCACAGCAACACCTGTTAGA
TRPM2	GGCAGCCTTGTACTTCAGTGAC	GAGGCAGAACAGGATGAAGTCC

TRPM3	GGAAAGGGCTCATCAAAGCAG	CCAACATGACGAATAACACCTGT
TRPM4	GCACGACGTTCATAGTTGACT	CTTCTCCGTGGTGTGTGCAT
TRPM5	GCCCACAGAGTGGACTTCATTGC	AGCTGGCTTCCCTCGCCAC
TRPM6	AACAGGAGCGTGGATAATAACTG	CAGACAGGATGAAGTGCGAGT
TRPM7	ACTGGAGGAGTAAACACAGGT	TGGAGCTATTCCGATAGTGCAA
TRPM8	GTGAAAGCGACTTGGTGAATTTT	GTGGCCTTGGAATCTTTGGTAA
TRPML1	TTCGCCGTCGTCTCAAATACT	CTCTTCCCGGAATGTCACAGC
TRPML2	GTTACGGCGAGGGCGAAATG	GCCATTGCATTTCTGACGGT
TRPML3	GAGTCGCTCGCTGACTCGC	GATCTGCCATCTCTGGGGGA
TRPP1	AGACCAACGATACCCTGTTCT	GGCTGTACCTCACTAGGACTC
TRPP2	TGACCTACGGCATGATGAGC	GTTTTCTCCGTTTTGGACACG
TRPP3	TGACAAGCTCCAGTGCTTATTAC	TCTGGTTGTTGTACCATTTGGTC
ATG5	AACTGAAAGGGAAGCAGAACCA	CCATTTCAGTGGTGTGCCTTC
LC3A	CCAGCAAAATCCCGGTGAT	TGGTCCGGGACCAAAAACT
LC3B	ACCATGCCGTCGGAGAAG	GGTTGGATGCTGCTCTCGAA
SQSTM1	GCCAGAGGAACAGATGGAGT	TCCGATTCTG GCATCTGTAG
